# A combination approach of behavioural and biomedical interventions for prevention of sexually transmitted infections

**DOI:** 10.2471/BLT.19.238170

**Published:** 2020-04-29

**Authors:** Igor Toskin, Nataliia Bakunina, Antonio Carlos Gerbase, Karel Blondeel, Rob Stephenson, Rachel Baggaley, Massimo Mirandola, Sevgi Okten Aral, Marie Laga, King Kennard Holmes, Christine Winkelmann, James Njogu Kiarie

**Affiliations:** a UNDP-UNFPA-UNICEF-WHO-World Bank Special Programme of Research, Development and Research Training in Human Reproduction (HRP), World Health Organization, avenue Appia 20, 1211 Geneva 27, Switzerland.; b Institute for Leadership and Health Management, I.M. Sechenov First Moscow State Medical University, Moscow, Russian Federation.; c Department of Systems, Population and Leadership, University of Michigan, Ann Arbor, United States of America (USA).; d HIV Department and Global Hepatitis Programme, World Health Organization, Geneva, Switzerland.; e Department of Diagnostics and Public Health, University of Verona, Verona, Italy.; f Centers for Disease Control and Prevention, Atlanta, USA.; g Institute of Tropical Medicine, Antwerp, Belgium.; h Department of Global Health, University of Washington, Seattle, Washington, USA.; i Federal Centre for Health Education (BZgA), Köln, Germany.

The World Health Organization (WHO) estimated that in 2016 the global annual incidence of chlamydia, gonorrhoea, trichomoniasis and syphilis among people 15 to 49 years of age was 376.4 million infections.^1^ The increased number of etiological pathogens known to be sexually transmissible, such as Zika and Ebola viruses, new outbreaks of acquired and congenital syphilis and *Lymphogranuloma venereum*, increasing antimicrobial resistance in *Neisseria gonorrhoeae* and potential resistance in other sexually transmitted infection pathogens, such as *Treponema pallidum* and *Mycoplasma genitalium*, raise additional concerns.^2^ Facing a global epidemic of sexually transmitted infections, the international public health agenda now emphasizes the importance of strengthening the control of such infections, including human immunodeficiency virus (HIV), through a combination prevention approach. This approach consists of the simultaneous use of rights-based, evidence-informed and complementary behavioural, biomedical and structural interventions operating at individual, relationship, community and societal levels. The combination of behavioural and biomedical approaches to sexually transmitted infections and/or HIV prevention is currently debated. Research and prevention programmes often give priority to biomedical approaches.^3^

Behavioural interventions have demonstrated effectiveness in reducing the incidence of sexually transmitted infections, including HIV, in various target groups and in a range of settings, whether these interventions are incorporated into combination prevention programmes or delivered separately.^4^^–^^6^ Several meta-analyses have shown that single-session behavioural interventions are as effective as more resource-consuming multisession intensive behavioural interventions for sexually transmitted infections and/or HIV transmission.^4^^,^^5^^,^^7^ Clearly, the findings differ across studies, varying in terms of intervention type and length, theoretical foundation, setting and study design. Most of the studies were conducted in the United States of America and other high-income countries, suggesting the need to examine the role of combining behavioural with biomedical interventions for the reduction of these infections in low- and middle-income countries (Fig. 1). 

**Fig. 1:**
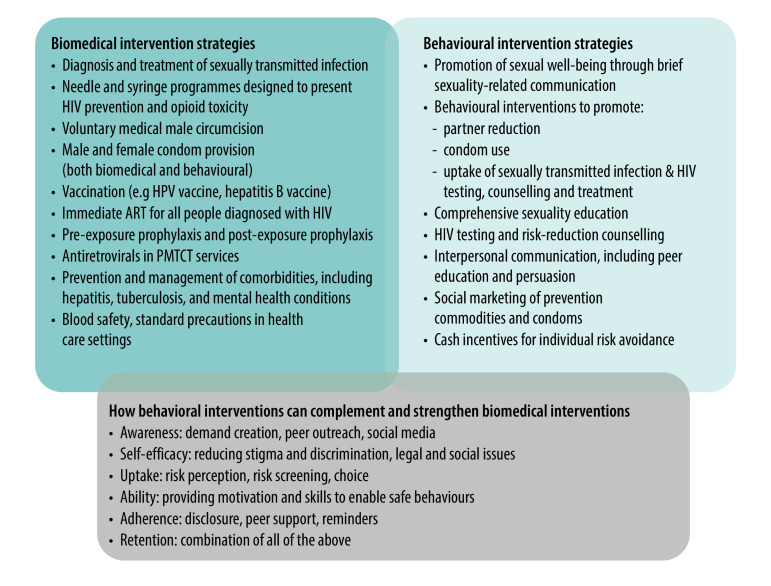
Biomedical and behavioural intervention strategies for combination prevention

ART: antiretroviral therapy; HIV: human immunodeficiency virus; HPV: human papillomavirus; PMTCT: prevention of mother-to-child transmission.

Source: *Consolidated guidelines on HIV prevention, diagnosis, treatment and care for key populations – 2016 update*.^3^

## Behavioural interventions

Before introducing behavioural interventions (including brief interventions), in the context of combination prevention programmes, the needs of different populations should be assessed, followed by the development of targeted programmes and interventions. Programmes will need to include skills training for providers, with a focus on shifting cultural and attitudinal norms where needed; and implementation and scale-up of the behavioural programmes, while monitoring and evaluating patients’ progress. Implementation should start with demonstration projects in settings where it is most feasible, and if successful, expanded to more challenging settings, engaging with antagonists, and using social and mass media to promote behavioural interventions.

**Fig. 2:**
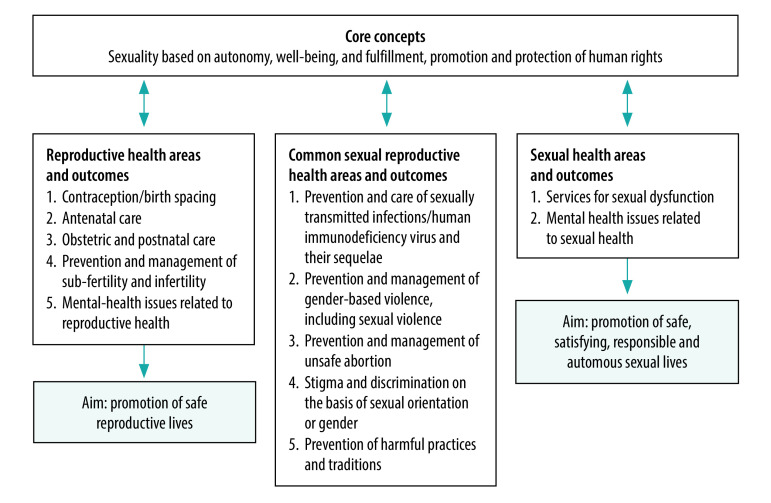
Core concepts underlying sexual and reproductive health

Source: Adapted from Hawkes.^8^

Positive attitudes towards sexuality, based on the core concepts of well-being (safety, autonomy and satisfaction) and promotion and protection of human rights, contribute to achieving positive health outcomes (Fig. 2).^8^ This notion is applicable to all thematic areas of sexual and reproductive health, including prevention and care of sexually transmitted infections and their sequelae, as agreed by stakeholders from all continents during the development of a conceptual framework for sexual health.^9^

In 2015, WHO recommended that policy-makers promote brief sexuality-related communication when possible, that is, a brief behavioural intervention with training for health-care providers to deliver such communication in diverse health-care settings. Brief sexuality-related communication is a clinical tool grounded in behaviour change theories; it is primarily based on motivational interviewing techniques, encompasses a holistic and positive understanding of sexual health and sexuality and addresses client-driven sexual health goals in a single session shorter than 25 minutes within primary health-care settings.^10^ Such communication has great potential if used as part of a combination prevention approach to reduce the incidence of sexually transmitted infections, including HIV, but wider experience is needed for implementation at the country level.^10^

## Challenges

Several barriers can hamper successful integration of behavioural interventions into existing services. Health systems are complex and introducing new strategies is not easy, especially when they relate to sexual issues and require additional contact between providers and clients. Strategic planning is often inadequate due to insufficient understanding of how an intervention works, how it fits into existing procedures and what synergies may exist with other interventions already in place. The economic implications of providing behavioural interventions and making them accessible must be considered to ensure that sexual health services are affordable. However, research on this cost is scarce. A recent literature review on the cost–effectiveness of prevention interventions of sexually transmitted infections in low- and middle-income countries yielded only one behavioural intervention study. That study assessed an online education programme for adolescents attending public schools in Colombia and concluded that the cost per sexually transmitted infection averted was between 95 and 824 United States dollars.^2^ Another systematic review summarized evidence from 60 studies on the cost–effectiveness of HIV prevention interventions in sub-Saharan Africa. That review included only one study assessing the impact of behaviour change, but none concerning impact of behavioural intervention.^11^ Numerous studies have assessed cost–effectiveness of various biomedical sexually transmitted infections and/or HIV prevention interventions, but estimates from implementation scenarios that consider local context, mixture of concurrent interventions, epidemics, individual adherence, time period and population, are still not available. Thus, further economic analysis and cost–effectiveness analyses are needed, comparing specific biomedical and behavioural interventions within a combination prevention approach where both interventions complement each other.

The effect sizes of behavioural interventions are complex to measure and mostly small when it comes to outcome indicators, such as decreased incidence of such infections. Furthermore, a lack of evidence on the persistence of intervention effects exists. More evidence needs to be generated on how to move from promising findings to scaling up interventions in resource-constrained settings within existing health systems using available resources (technical expertise and human, physical and financial resources). The main issue with the current research on behavioural interventions to prevent sexually transmitted infections and/or HIV is the lack of a common framework within which to assess why and when something may or may not work. Determining the active component of such interventions could provide a basis for designing reproducible and more effective interventions.

There is often a lack of connection between those who recommend behavioural interventions and the government health departments responsible for their implementation . Support and guidance on how to roll out behavioural interventions to prevent these infections are desirable, as are experience, training and skills of the health-care providers in delivery of brief behavioural interventions and in discussing topics around sexuality.^10^ Brief sexuality-related communication guidelines recommend this approach for primary health services and training of health-care providers in sexual health knowledge and communication.^10^

## The way forward

Box 1 lists ways to support successful introduction of behavioural interventions in the context of the combination prevention approach. The key novel element is training of health-care providers grounded in the understanding of the concept of healthy sexuality. A standardized brief sexuality-related communication intervention and relevant training for providers, consistent with related guidelines, is currently being piloted in Peru and the Republic of Moldova, and adapted to the local contexts and target population needs using qualitative methods.^12^ The principles of motivational interviewing have been also widely implemented in the mental health field, particularly for disorders due to substance use. The objective of universal health coverage provides a favourable platform for integration of such interventions. Health promotion to encourage healthy diets, more physical activity and regular early screening, and smoking and alcohol consumption cessation, often with the use of digital technologies, is a common approach to noncommunicable disease prevention. This approach creates an enabling environment for combining the efforts within primary prevention programmes in noncommunicable and communicable diseases, including sexually transmitted infections, where motivational interviewing aimed at behaviour change towards a healthy lifestyle could be a universal communication tool between providers and clients.

Box 1 **Key priorities to advance behavioural interventions for sexually transmitted infections and/or HIV prevention and reduction**

- Train health-care providers in communication and culturally sensitive competence around sexuality.
- Develop an intervention manual and guidelines, including provider training, on behavioural interventions and brief sexuality-related communication in particular.
- Tailor behavioural interventions to various target groups through close collaboration with the target population and their health-care providers.
- Gain understanding of the technical capacity of health-care providers and their supervisors to integrate new activities at their health centres.
- Simplify all interventions for easier integration into existing services and systems.
- Make interventions affordable and more accessible.
- Strategically link biomedical and (brief) behavioural interventions in the process of planning and implementation.
- Engage decision-makers, civil society groups and all gatekeepers, from the policy level to the facility and community level, in the initial stages of programme development and implementation.
- Take advantage of opportunities to use information and technologies and mobile health applications to reduce, though not replace, or complement the time required for human interaction with a client.
- Set up appropriate study designs, including randomized controlled trials and mixed-method studies, and disaggregate the data on each component or active ingredient of each intervention, to examine what is or is not actually effective.
- Develop, evaluate and promote behavioural interventions in a way that is linked to the currently available and forthcoming biomedical interventions (pre-exposure prophylaxis; circumcision; point-of-care, community-based and self-administered testing; and multi-purpose prevention technologies).^a^

HIV: human immunodeficiency virus.^a^ Multipurpose prevention technologies are products that provide protection against both unintended pregnancy and sexually transmitted infections, including HIV.

In general, the combination prevention approach could benefit from the promotion of sexual health as a pivotal public health concept. Long-term outcomes of the proposed strategy will include a paradigm shift from a disease-centred approach to sexual well-being, to better trained health-care providers with a positive attitude towards sexuality and tailored behavioural interventions linked to biomedical approaches in one package to prevent sexually transmitted infections.

## Conclusion

Brief behavioural interventions delivered in addition to and/or as part of a package with biomedical interventions are essential components of effective strategies for sexually transmitted infections prevention. Complementary behavioural and biomedical interventions form a comprehensive combination prevention solution that can increase the positive impact on sexual health, including the decrease of these infections. Behavioural interventions will only be effective if their design is evidence-informed and tailored to the target groups, local settings and cultural contexts, and if health-care providers are sufficiently trained. The content of the intervention and the training should be based on the personal and social aspects of human sexuality. Therefore, additional evidence on the feasibility of new and promising behavioural interventions, and procedural guidelines for effective delivery and implementation of behavioural interventions, are needed. Strategic cooperative actions will allow the addition of these behavioural tools to constructively complement the biomedical solutions that are already employed, to more successfully control sexually transmitted infections and HIV epidemics.
